# Guy's Hospital Medical School

**Published:** 1913-06-07

**Authors:** 


### GUY'S HOSPITAL MEDICAL SCHOOL.

### The New Buildings of the Medical and Dental Schools Described.

The completed buildings of Guy's Hospital Medical and Dental School, which Mr. Balfour opened on Tuesday last, are the result of ten years' work. They consist of three main blocks, the block containing the Department of Physiology, the new dental block, which was completed last year, and the block containing the Department of Pathology. Taking these in turn we find that the first, which stands on the west side of the park and faces Hunt's House, comprises an entrance hall with busts of famous Guy's men, like Dr. Addison or Sir Astley Cooper. On the left is the students' laboratory for physiological chemistry, and a large lecture theatre accommodating 400 students, while on the right are the research laboratories of the demonstrators. In the basement beneath are the *x*-ray rooms, certain class-rooms and store-rooms. On the ground floor also is the Wills Library, with at its end the room set apart as a museum of Guy's antiquities. Here also is the Gordon Museum, which was presented to the school eight years ago by Mr. Robert Gordon. This museum contains the famous wax models made by the late Mr. Towne, and is divided into four compartments, round each of which run two galleries, and the shelves accommodate some ten thousand specimens. Climbing up to the first floor we come to the Students' Histological Laboratory and the research laboratories of the lecturer in physiology; on the left are the offices of the Medical School and the staff's committee-room. The second floor comprises the Department of Physics. Here, in other words, are the students' laboratory for experimental physics, a dark-room, class-rooms, and the laboratories of the lecturers on physics and chemistry. The lectures in these subjects are delivered in a large lecture theatre on this floor. Above, on the third and top floor, are the students' chemical laboratory, with a balance-room, and, again, a research room for the demonstrators. There is also a smaller laboratory for chemical work of the Department of Public Health. This completes a brief summary of the main block of the new buildings, and it will be seen from this how comprehensive they are.

Not less important is the new dental block, another building, also three storeys in height, which surround a central courtyard. Students and patients have separate entrances, and on the ground floor are a waiting and cloak room, the resident dental officer's room, and the laboratory for probationers' work. Above, on the first floor, are the students' mechanical laboratory, where an apprenticeship is served; the pupils' mechanical laboratory, equipped for 100 pupils, with all the electric and other apparatus required for the teaching of prosthenic dentistry. On the first floor also is the metallurgical laboratory, with two muffle furnaces for assaying gold and silver, eight injector gas furnaces for preparing amalgam and other alloys, all of which are operated by an electric mechanical blower. From the general public's point of view the "show" room of the dental block is the conservation-room, in which eighty pump chairs are placed in rows on all the four sides of the central courtyard. Its total area is about 6,000 feet.

The Department of Pathology is the last of the three main blocks of the completed buildings. It is five storeys high. It stands behind the Dental School in necessary relation to the adjacent mortuary and post-mortem room. The whole building is devoted to teaching and research. The sum of £2,000 was expended on its equipment, which an anonymous donor presented free of charge to the hospital. The authorities remark that: No part of the building is used for the routine pathological work of the hospital, but the whole is devoted to teaching and research. The routine pathological, histological, and bacteriological work of the hospital, necessary for clinical investigations and the treatment of patients, is carried on by other workers in separate laboratories at the expense of the hospital. On the first floor are the laboratories for teaching morbid histology and bacteriology; on the second are the pharmacological laboratories; on the third are the private laboratories; and the fourth is given up to the experimental work of the Gordon Lecturer in Pathology.

Such, in brief summary, are the principal features of the new Medical and Dental Schools. Over £100,000 has been spent upon them, and they now claim to be the equals of the medical schools of the larger provincial universities. Of the sum spent no less than £77,000 has been found by the staff, the governors, and their friends.

The buildings have been completed and in part paid for, but a further sum of £200,000 is necessary before the work of investigation and the prosecution of new methods of treatment can be carried on at the utmost pressure.

